# Rubicon-regulated beta-1 adrenergic receptor recycling protects the heart from pressure overload

**DOI:** 10.1038/s41598-021-03920-6

**Published:** 2022-01-07

**Authors:** Yasuhiro Akazawa, Manabu Taneike, Hiromichi Ueda, Rika Kitazume-Taneike, Tomokazu Murakawa, Ryuta Sugihara, Hiroki Yorifuji, Hiroki Nishida, Kentaro Mine, Ayana Hioki, Shigemiki Omiya, Hiroyuki Nakayama, Osamu Yamaguchi, Tamotsu Yoshimori, Yasushi Sakata, Kinya Otsu

**Affiliations:** 1 Department of Cardiovascular Medicine, Osaka University Graduate School of Medicine, 2-2 Yamadaoka, Suita, Osaka 565-0871 Japan; 2 Preventive Diagnostics, Department of Biomedical Informatics, Division of Health Sciences, Osaka University Graduate School of Medicine, 1-7 Yamadaoka, Suita, Osaka 565-0871 Japan; 3 The School of Cardiovascular Medicine and Sciences, King’s College London British Heart Foundation Centre of Excellence, 125 Coldharbour Lane, London, SE5 9NU UK; 4 Department of Cardiology, Pulmonology, Hypertension and Nephrology, Ehime University Graduate School of Medicine, 454 Shitsukawa, Toon, Ehime 791-0295 Japan; 5 Department of Genetics, Osaka University Graduate School of Medicine, 2-2 Yamadaoka, Suita, Osaka 565-0871 Japan

**Keywords:** Molecular biology, Cardiology

## Abstract

Heart failure has high morbidity and mortality in the developed countries. Autophagy is important for the quality control of proteins and organelles in the heart. Rubicon (Run domain Beclin-1-interacting and cysteine-rich domain-containing protein) has been identified as a potent negative regulator of autophagy and endolysosomal trafficking. The aim of this study was to investigate the in vivo role of Rubicon-mediated autophagy and endosomal trafficking in the heart. We generated cardiomyocyte-specific Rubicon-deficient mice and subjected the mice to pressure overload by means of transverse aortic constriction. Rubicon-deficient mice showed heart failure with left ventricular dilatation, systolic dysfunction and lung congestion one week after pressure overload. While autophagic activity was unchanged, the protein amount of beta-1 adrenergic receptor was decreased in the pressure-overloaded Rubicon-deficient hearts. The increases in heart rate and systolic function by beta-1 adrenergic stimulation were significantly attenuated in pressure-overloaded Rubicon-deficient hearts. In isolated rat neonatal cardiomyocytes, the downregulation of the receptor by beta-1 adrenergic agonist was accelerated by knockdown of Rubicon through the inhibition of recycling of the receptor. Taken together, Rubicon protects the heart from pressure overload. Rubicon maintains the intracellular recycling of beta-1 adrenergic receptor, which might contribute to its cardioprotective effect.

## Introduction

Heart failure is the final stage of most heart diseases, such as idiopathic and ischemic cardiomyopathies and valvular diseases and is one of the major causes of death in the developed countries^1^. Thus, there is a need to elucidate the molecular mechanism of the development and progression of heart failure and to find a novel therapeutic target for this disease.

Autophagy is one of the intracellular systems for degrading unnecessary proteins and damaged organelles and also has an important role in the quality control of proteins and organelles to maintain cellular homeostasis. Our loss-of-function studies have shown that autophagy protects the heart against pressure overload and aging^2,3^. To confirm the cardioprotective role of autophagy during cardiac remodeling, the effect of autophagy activation on cardiac function should be examined. Rubicon (Run domain Beclin-1-interacting and cysteine-rich domain-containing protein) was identified as a negative regulator of autophagy via the inhibition of autophagosome and lysosome fusion^4^. An in vitro study indicates that Rubicon is also a negative regulator of endosomal degradation by inhibiting the fusion of endosomes with lysosomes^5^. In tissue-specific Rubicon-deficient mice, the development of nonalcoholic fatty liver disease was suppressed^6^, aging phenotypes in the kidney and the brain^7^ via the stimulation of autophagic activity were reduced, and cardiac ischemia/reperfusion injury was attenuated^8^, indicating the detrimental effect of Rubicon. On the other hand, kidney-specific Rubicon-deficient mice developed metabolic syndrome^9^, indicating its protective effect. These data^6,7,9^ suggest that the role of Rubicon might depend on the tissue and experimental conditions in vivo.

The $\upbeta$1 adrenergic receptor, one of the major G protein-coupled receptors, is localized on the cell surface of cardiomyocytes. The binding of ligands released into circulation under various conditions, such as exercise, hypertension and valvular dysfunction, with the receptor induces a variety of biological reactions in the heart: increased beating rate, contractile force, and relaxation speed, and cardiac hypertrophy^10^. Those compensated reactions are necessary for maintaining the cardiac output and structure against stress. However, sustained stimulation of the receptor by the ligands shifts the heart from the compensated to decompensated phase, shown as cardiac chamber dilatation and dysfunction. The maladaptive responses are accompanied by the desensitization and internalization of $\upbeta$1 adrenergic receptors. The receptor is internalized into the cytosol by endocytosis, and is degraded by fusion with the lysosome or is translocated back from the endosome onto the cell membrane (recycling)^11^. However, the regulatory mechanism for the degradation of the internalized receptor remains unclear.

In the present study, we aimed to elucidate the role of Rubicon in pressure-overloaded mouse hearts. We found that Rubicon protects the heart and that the maintenance of the intracellular recycling of $\upbeta$1 adrenergic receptor might contribute to the cardioprotective effect of Rubicon through negative regulation of endocytic degradation.

## Results

### Cardiomyocyte-specific ablation of Rubicon led to the development of heart failure in response to pressure overload

**Figure 1:**
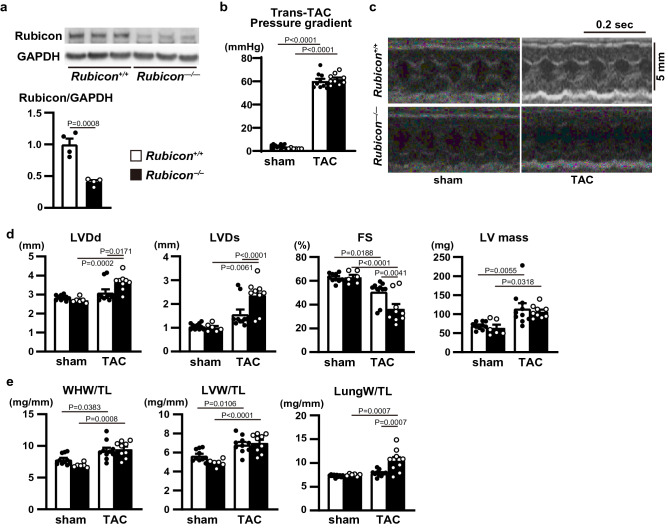
Cardiac phenotypes of cardiomyocyte-specific Rubicon-deficient mice after pressure overload. (**a**) Knockout efficiency of Rubicon in *Rubicon*^−/−^ hearts at baseline. LV homogenates from *Rubicon*^+/+^ and *Rubicon*^−/−^ mice were subjected to western blot analysis (N = 4). Student’s *t*-test was used for analysis. A cropped blot is displayed and a full-length blot is presented in Supplementary Fig. S3a. (**b**) Trans-TAC pressure gradient was estimated from the difference of blood pressure between upper limbs. (**c**,**d**) Trans-thoracic M-mode echocardiographic analysis one week after transverse aortic constriction (TAC). Representative images are shown in (**c**). Scale bars: 0.2 s and 5 mm, respectively. Echocardiographic measurements are shown in (**d**). LVDd, end-diastolic internal dimension of left ventricle (LV); LVDs, end-systolic internal dimension of LV; FS, fractional shortening of LV dimension. (**e**) Physiological parameters one week after TAC. WHW, whole heart weight; TL, tibia length; LVW, left ventricle weight; LungW, lung weight. N = 10 (sham-*Rubicon*^+/+^), 6 (sham-*Rubicon*^−/−^), 10 (TAC-*Rubicon*^+/+^) or 9 (TAC-*Rubicon*^−/−^) and one-way ANOVA for analysis was used in (**b**) and (**c**). Open and closed bars indicate *Rubicon*^+/+^ and *Rubicon*^−/−^, respectively. Data are expressed as the mean ± s.e.m.

**Figure 2:**
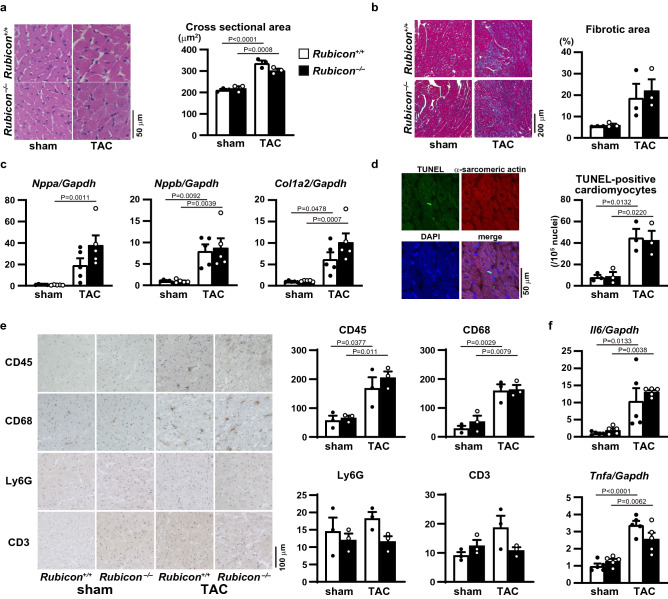
Hypertrophic and inflammatory responses in TAC-operated *Rubicon*^−/−^ hearts. (**a**) Representative hematoxylin–eosin-stained sections of *Rubicon*^+/+^ and *Rubicon*^−/−^ hearts. Right panel, quantitative analysis of cross-sectional area of cardiomyocytes. Scale bar: 50 $\upmu$ m. N = 3. (**b**) Representative Azan-Mallory-stained sections of the hearts. Right panel, quantitative analysis of fibrotic area. Scale bar: 200 $\upmu$ m. N = 3. (**c**) Expression levels of mRNAs related to cardiac remodeling. Data were normalized to the *Gapdh* content and are expressed as fold increase over levels in the sham-operated *Rubicon*^+/+^ group. N = 5. (**d**) Representative confocal images of TUNEL-positive cardiomyocytes. Right panel, quantitative analysis of TUNEL-positive cardiomyocytes. N = 3. (**e**) Representative images of immunohistochemical analysis of CD45, CD68, Ly6G and CD3. Scale bar: 100 $\upmu$ m. Right graphs show quantitative analysis of each infiltrating inflammatory cell type. N = 3. (**f**) Expression levels of cytokine mRNAs. Data were normalized to the *Gapdh* content and are expressed as fold increase over levels in sham-operated *Rubicon*^+/+^ group. N = 5. Open and closed bars indicate *Rubicon*^+/+^ and *Rubicon*^−/−^, respectively. Data are expressed as the mean ± s.e.m. One-way ANOVA was used for analysis.

To investigate the in vivo role of Rubicon in the heart, we generated cardiomyocyte-specific Rubicon-deficient mice (*Rubicon*^−/−^) by crossing floxed Rubicon mice^6^ with transgenic mice expressing *Cre* recombinase under the control of the $\mathrm{\alpha }$-myosin heavy chain promoter ($\mathrm{\alpha }$-MHC)^12^. Their littermates (*Rubicon*^+/+^) were used as the control. The *Rubicon*^−/−^ mice were born at the expected Mendelian frequency (male *Rubicon*^+/+^ : male *Rubicon*^−/−^ : female *Rubicon*^+/+^ : female *Rubicon*^−/−^  = 20:18:21:20) and were indistinguishable from their littermates. The mice grew to adulthood and showed normal fertility. Although the protein level of Rubicon in the *Rubicon*^−/−^ hearts was significantly reduced (Fig. 1a), the mice did not have significant differences in echocardiographic (Table 1) and physiological (Table 2) parameters at 10 weeks of age compared to the littermates. These data indicate that Rubicon has no significant influence on the global cardiac structure and function under normal conditions in the heart up to 10 weeks of age. Then, the mice were subjected to pressure overload by means of transverse aortic constriction (TAC) surgery^13^ to examine the in vivo role of Rubicon during cardiac remodeling. Initially, ablation of Rubicon was expected to induce upregulated autophagic activity and to protect the heart from hemodynamic stress, as we have previously reported^2,4^. However, both TAC-operated *Rubicon*^−/−^ and *Rubicon*^+/+^ mice exhibited reduced fractional shortening of left ventricular (LV) dimension (FS), an indicator of cardiac systolic function, one week after TAC (Fig. 1b,c,d). The *Rubicon*^−/−^ mice showed more severe cardiac dysfunction and LV chamber dilation than the *Rubicon*^+/+^ mice. After TAC, the lung weight-to-tibia length ratio, an indicator of lung congestion, was significantly increased in the *Rubicon*^−/−^ mice, but not in the *Rubicon*^+/+^ mice, indicating LV heart failure in the *Rubicon*^−/−^ mice (Fig. 1e). Although LV mass and the ratios of whole heart weight and LV weight to tibia length, indicators of cardiac hypertrophy, were elevated in both groups of TAC-operated mice, there was no difference between the two groups (Fig. 1d,e). Histological analyses revealed that the cross-sectional area of the cardiomyocytes was increased and that the fibrotic area tended to be increased by TAC in both groups, but did not show differences between the two groups (Fig. 2a,b). The mRNA expression levels of *Nppa* (atrial natriuretic peptides), *Nppb* (brain natriuretic peptides) and *Col1a2* (collagen type I $\mathrm{\alpha }$ 2) were not different between the two TAC-operated groups (Fig. 2c). Both TAC-operated groups also increased the numbers of apoptotic cardiomyocytes, evaluated with TUNEL (terminal deoxynucleotidyl transferase-mediated dUTP-biotin nick end labeling) assay (Fig. 2d), and infiltrated inflammatory cells such as CD45- and CD68-positive cells (Fig. 2e) and inflammatory cytokine mRNA levels (Fig. 2f), but there were no differences between the two groups. Contrary to our hypothesis, these data suggest that the ablation of Rubicon in cardiomyocytes resulted in heart failure in response to pressure overload without affecting the hypertrophic responses, apoptosis or inflammation.

**Table 1:** Echocardiographic parameters of *Rubicon*^*−/−*^ mice.

	*Rubicon*^+*/*+^ (n = 11)	*Rubicon*^*−/−*^ (n = 12)
IVSd (mm)	0.75 ± 0.02	0.80 ± 0.02
LVDd (mm)	2.76 ± 0.03	2.82 ± 0.05
LVDs (mm)	1.20 ± 0.03	1.24 ± 0.03
LVPWd (mm)	0.74 ± 0.02	0.80 ± 0.02
FS (%)	57.5 ± 1.1	55.9 ± 0.6
HR (bpm)	704 ± 11	712 ± 9

IVSd, end-diastolic interventricular septal wall thickness; LVDd, end-diastolic internal dimension of left ventricle (LV); LVDs, end-systolic internal dimension of LV; LVPWd, end-diastolic LV posterior wall thickness; FS, fractional shortening of LV dimension; HR, heart rate. Student’s *t*-test was used. There were no significant differences between groups. Values represent the mean ± s.e.m. of data.

**Table 2:** Physiological parameters of *Rubicon*^*−/−*^ mice.

	*Rubicon*^+*/*+^ (n = 9)	*Rubicon*^*−/−*^ (n = 9)
Blood pressure (mmHg)	72 ± 3	72 ± 3
Body weight (g)	25.7 ± 0.6	25.7 ± 0.2
Tibia (mm)	17.5 ± 0.1	17.5 ± 0.1
WH/Tibia (mg/mm)	6.82 ± 0.13	6.92 ± 0.11
LV/Tibia (mg/mm)	4.65 ± 0.10	4.85 ± 0.08
Lung/Tibia (mg/mm)	7.06 ± 0.14	7.10 ± 0.06

WH/Tibia, whole heart weight-to-tibia length ratio; LV/Tibia, left ventricle weight-to-tibia length ratio; Lung/Tibia, lung weight-to tibia length ratio. Student’s *t*-test was used. There were no significant differences between groups. Values represent the mean ± s.e.m. of data.

### $\upbeta$1 adrenergic receptor was decreased in ***Rubicon***^−/−^ hearts without upregulation of autophagic activity after TAC

**Figure 3:**
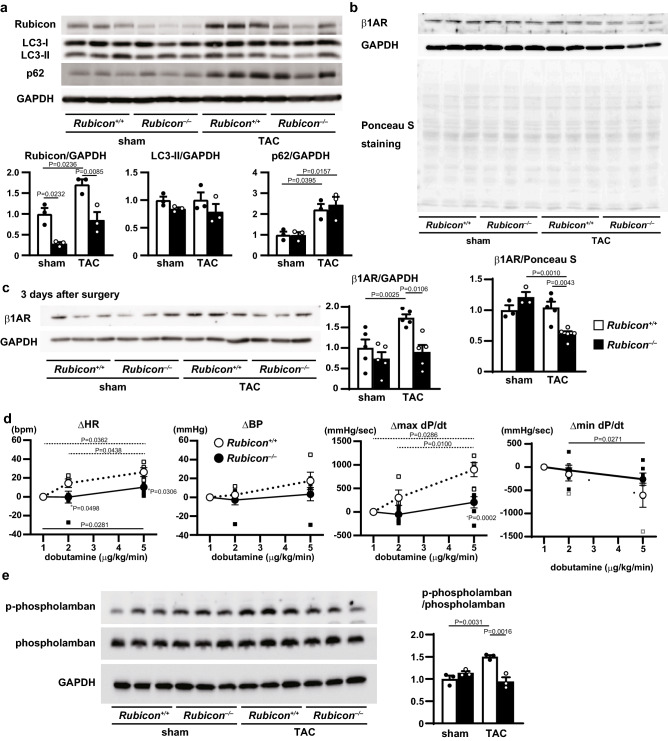
Decreases in $\upbeta$1 adrenergic receptor and responses to $\upbeta$1 stimulation in TAC-operated *Rubicon*^−/−^ hearts. (**a**) Western blot analyses of the autophagic markers, Rubicon, LC3 and p62, in the hearts. Bottom panels, quantitative analyses. N = 3 per group. (**b**) Western blot analysis of $\upbeta$1 adrenergic receptor (AR). Bottom panel, quantitative analysis. Signals from Ponceau S staining were used for normalization. N = 3 (sham-*Rubicon*^+/+^), 3 (sham-*Rubicon*^−/−^), 5 (TAC-*Rubicon*^+/+^) or 5 (TAC-*Rubicon*^−/−^). A one-way ANOVA was used for analysis. Open and closed bars indicate *Rubicon*^+/+^ and *Rubicon*^−/−^, respectively. (**c**) Western blot analysis of $\upbeta$1AR 3 days after TAC. Right panel, quantitative analysis. N = 3. A one-way ANOVA was used for analysis. Open and closed bars indicate *Rubicon*^+/+^ and *Rubicon *^−/−^, respectively. *P < 0.0001 versus all the others. (**d**) Cardiac function measured with a catheter during dobutamine treatment. Values express differences of the following parameters between each point and 1 $\upmu$g/kg/min dobutamine; heart rate (HR), end-systolic left ventricular blood pressure (BP), the maximal value of the first derivative of left ventricular pressure (max dP/dt), the minimal value of the first derivative of left ventricular pressure (min dP/dt). N = 4 (*Rubicon*^+/+^) or 6 (*Rubicon*^−/−^). A repeated measure two-way ANOVA was used for analysis. Solid and dotted lines indicate a significant difference between two different concentrations of dobutamine in the *Rubicon*^+/+^ and *Rubicon*^−/−^ hearts, respectively. *P < 0.05 versus corresponding *Rubicon*^+/+^ group. Open and closed circles indicate *Rubicon*^+/+^ and *Rubicon*^−/−^, respectively. Data are expressed as the mean ± s.e.m. (**e**) Western blot analysis of phospho-phospholamban. Data are normalized to phospholamban. The average value for sham-operated *Rubicon*^+/+^ mice was set to 1. Right panel, quantitative analysis. N = 3. A one-way ANOVA was used for analysis. Open and closed bars indicate *Rubicon*^+*/*+^ and *Rubicon*^*−/−*^, respectively. Cropped blots are displayed and full-length blots are presented in Supplementary Fig. S3b-e.

To investigate the mechanism of the phenotypes observed in the *Rubicon*^−/−^ mice, the autophagic activity in the heart was evaluated with western blotting (Fig. 3a). One week after the operation, the sham- or TAC-operated *Rubicon*^−/−^ hearts had lower protein levels of Rubicon than the corresponding *Rubicon*^+/+^ hearts. In addition, TAC increased the Rubicon protein level in the *Rubicon*^+/+^ hearts. However, the protein level of microtubule associated protein light chain 3 (LC3)-II, a marker of autophagic activity, was not different among all groups. Furthermore, p62 protein level, another marker of autophagic activity, was increased in both TAC-operated groups, but was not different between the two groups. These data suggest that the heart failure observed in the TAC-operated *Rubicon*^−/−^ mice was not due to the alteration of autophagic activity. As down-regulation of $\upbeta$1 adrenergic receptor is one of the causes of heart failure^10^, we examined the expression level of $\upbeta$1 adrenergic receptor with western blotting. Figure 3b shows that the level of $\upbeta$1 adrenergic receptor was significantly reduced in the TAC-operated *Rubicon*^−/−^ hearts compared to the TAC-operated *Rubicon*^+/+^ hearts or sham-operated *Rubicon*^−/−^ hearts one week after operation. There was no significant difference in the transcript levels of $\upbeta$1 adrenergic receptor between *Rubicon*^+/+^ and *Rubicon*^−/−^ groups at baseline (Supplementary Fig. S1a).

### Responses to $\upbeta$1 stimulation were attenuated in TAC-operated ***Rubicon***^−/−^ hearts

To investigate the effect of Rubicon deficiency on the development of heart failure, we analyzed the mice three days after TAC to minimize the secondary effect of heart failure. Both TAC-operated groups did not show any LV chamber dilatation or dysfunction at that time point (Table 3). Western blot analysis showed the expression level of $\upbeta$1 adrenergic receptor was downregulated in *Rubicon*^−/−^ hearts compared to that in *Rubicon*^+/+^ hearts (Fig. 3c). Then, we examined cardiac function in the mice using a catheter (Fig. 3d). Dobutamine infusion at 5 $\upmu$g/kg/min increased heart rate (HR) significantly compared to that at 1 $\upmu$g/kg/min dobutamine infusion in both *Rubicon*^+/+^ and *Rubicon*^−/−^ hearts; however, the HR was significantly lower in the *Rubicon*^−/−^ hearts than in the *Rubicon*^+/+^ hearts. The maximum dP/dt (maximal value of the first derivative of LV pressure), an indicator of systolic function, was significantly increased at 5 $\upmu$g/kg/min dobutamine infusion compared to that at 1 $\upmu$g/kg/min dobutamine infusion in the *Rubicon*^+/+^ hearts, but not in the *Rubicon*^−/−^ hearts. Blood pressure and minimum dP/dt also showed a similar tendency. These data suggest less responsiveness against $\upbeta$ stimulation in *Rubicon*^−/−^ than *Rubicon*^+/+^ hearts. In addition, we performed western blotting analysis to evaluate the expression level of phospholamban, one of excitation–contraction coupling proteins, in the hearts 3 days after TAC, and showed a decrease in the level of phosphorylated phospholamban in TAC-operated KO hearts (Fig. 3e). The decreases in $\upbeta$1 adrenergic receptor and inotropic responses could be a cause of the cardiac dysfunction shown in the *Rubicon*^−/−^ hearts after TAC.

**Table 3:** Echocardiographic parameters of *Rubicon*^*−/−*^ mice 3 days after TAC.

	Sham		TAC	
	*Rubicon*^+*/*+^ (n = 7)	*Rubicon*^*−/−*^ (n = 6)	*Rubicon*^+*/*+^ (n = 10)	*Rubicon*^*−/−*^ (n = 6)
IVSd (mm)	0.69 ± 0.04	0.63 ± 0.04	0.72 ± 0.04	0.75 ± 0.03
LVDd (mm)	2.51 ± 0.08	2.57 ± 0.08	2.72 ± 0.07	2.71 ± 0.06
LVDs (mm)	0.95 ± 0.06	0.89 ± 0.04	0.99 ± 0.04	1.03 ± 0.07
LVPWd (mm)	0.63 ± 0.02	0.63 ± 0.03	0.78 ± 0.05	0.73 ± 0.03
FS (%)	62.5 ± 1.4	65.4 ± 1.2	63.8 ± 1.3	62.1 ± 2.8
HR (bpm)	694 ± 15	693 ± 7	696 ± 14	710 ± 13

IVSd, end-diastolic interventricular septal wall thickness; LVDd, end-diastolic internal dimension of left ventricle (LV); LVDs, end-systolic internal dimension of LV; LVPWd, end-diastolic LV posterior wall thickness; FS, fractional shortening of LV dimension; HR, heart rate. One-way ANOVA followed by Tukey’s post hoc test was used. There were no significant differences between groups. Values represent the mean ± s.e.m. of data.

### Rubicon regulated $\upbeta$1 adrenergic receptor recycling under $\upbeta$1 adrenergic stimulation

**Figure 4:**
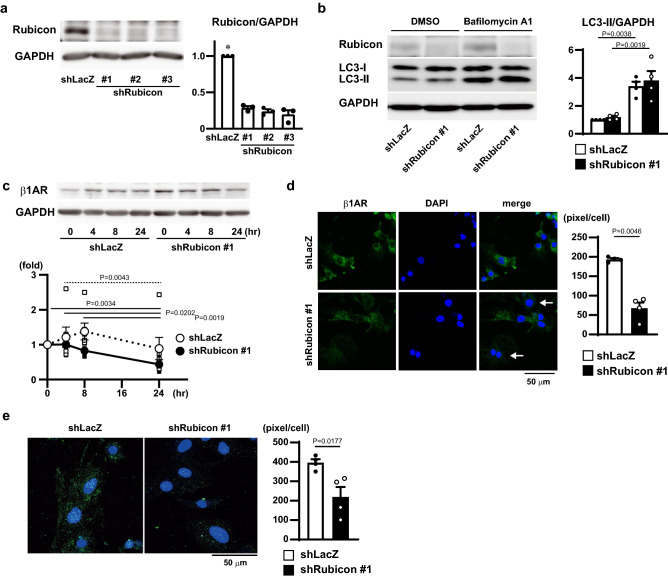
Rubicon-regulated $\upbeta$1 adrenergic receptor recycling under $\upbeta$1 adrenergic stimulation. (**a**) Western blot analysis of Rubicon in neonatal rat cardiomyocytes (NRCMs) infected with three different kinds of adenovirus expressing shRNA targeted to *Rubicon* mRNA (shRubicon). Right panel, quantitative analysis of Rubicon. Data were normalized to GAPDH and are expressed as fold increase over levels in NRCM infected with adenovirus expressing shRNA targeted to LacZ mRNA (shLacZ). N = 3. One-way ANOVA was used for analysis. *P < 0.0001 versus all the others. (**b**) Immunoblots for LC3. NRCMs infected with adenovirus expressing shLacZ or shRubicon were treated with or without 100 nM of Bafilomycin A1. Right panel, quantitative analysis of LC3-II. Data were normalized to GAPDH and the value for cells infected with shLacZ and treated with DMSO in each experiment was set equal to 1. N = 4. A one-way ANOVA was used for analysis. Open and closed bars indicate shLacZ and shRubicon, respectively. (**c**) Western blot analysis of $\upbeta$1 adrenergic receptor (AR) in NRCMs infected with shRubicon after isoproterenol treatment. Lower panel, quantitative analysis of $\upbeta$1AR. Data were normalized to GAPDH and are expressed as fold increase over levels in corresponding 0 h. N = 6. A non-repeated measure two-way ANOVA was used for analysis. Solid and dotted lines indicate significant differences between two different time points in shLacZ and shRubicon groups, respectively. Open and closed circles indicate, shLacZ and shRubicon #1, respectively. Cropped blots are displayed and full-length blots are presented in Supplementary Fig. S4. (**d**) Representative confocal images of recycling assay. Staining for $\upbeta$1AR is shown in green, and that for DAPI in blue. Arrows indicate NRCMs with weak green signal. Scale bar, 50 $\upmu$m. Right panel, quantitative analysis of $\upbeta$1AR-positive signal in cells. N = 4. Student’s *t*-test was used for analysis. Open and closed bars indicate, shLacZ and shRubicon, respectively. Data are expressed as the mean ± s.e.m. (**e**) Intracellular $\upbeta$1AR was analyzed by confocal microscope. Staining for $\upbeta$1AR is shown in green, and that for DAPI in blue. Scale bar, 50 $\upmu$m. Right panel, quantitative analysis of $\upbeta$1AR-positive signal in cells. N = 4. Student’s *t*-test was used for analysis. Open and closed bars indicate, shLacZ and shRubicon, respectively. Data are expressed as the mean ± s.e.m.

To investigate the mechanism for the Rubicon-mediated decrease in $\upbeta$1 adrenergic receptor in vitro, neonatal rat cardiomyocytes (NRCM) were infected with adenoviral vectors expressing short hairpin RNA (shRNA) targeted to Rubicon. The infection resulted in > 70% reduction in the protein expression level of Rubicon (Fig. 4a). Knockdown of Rubicon did not change autophagic flux (Fig. 4b) and the transcript level of $\upbeta$1 adrenergic receptor (Supplementary Fig. S1b). Then, NRCM infected with the shRNA adenoviral vector to Rubicon (shRubicon) were incubated with a $\upbeta$1 adrenergic receptor agonist, isoproterenol (1 $\upmu$M), accompanied by cycloheximide (10 $\upmu$g/ml), a translation inhibitor, for 24 h. Figure 4c shows that the shRubicon-infected cells significantly decreased the protein levels of $\upbeta$1 adrenergic receptor 24 h after stimulation compared to baseline, but the control shLacZ (shRNA targeted to *LacZ* mRNA)-infected cells did not.

To evaluate the level of $\upbeta$1 adrenergic receptor recycling in the cells, a recycling assay was performed. To label the internalized $\upbeta$1 adrenergic receptor, the cells were incubated with an anti-$\upbeta$1 adrenergic receptor antibody for one hour and washed. After further 1 h incubation, the receptor recycled onto the cell membrane was detected with a fluorescence-conjugated secondary antibody. Significantly less immunofluorescence signal was detected in Rubicon-knockdown cells (Fig. 4d), suggesting that the knockdown of Rubicon resulted in a decrease in the amount of recycled $\upbeta$1 adrenergic receptor. Immunocytochemical analysis showed that the amount of intracellular $\upbeta$1 adrenergic receptor was also decreased in Rubicon-knockdown cells (Fig. 4e).

## Discussion

Our data indicate that Rubicon does not contribute significantly to cardiac hypertrophic responses, inflammation, cell death or the autophagic pathway, but suggest its possible role to protect hearts against pressure overload by maintaining $\upbeta$1 adrenergic receptor. However, we failed to present direct evidence showing that impaired cardiac function in TAC-operated *Rubicon*^–/–^ mice was a consequence of reduced β1 adrenergic receptor. This is the first report showing a part of the $\upbeta$1 adrenergic receptor recycling mechanisms in failing hearts.

The ablation of positive regulators of autophagy in the heart, such as Becline 1 and Atg5 ^2,14^, induced heart failure with a decrease in autophagic activity, suggesting the protective role of autophagy during cardiac remodeling. Initially, we hypothesized that the ablation of Rubicon would accelerate autophagic activity, preventing the development of heart failure after TAC. However, the autophagic activity was not different between the *Rubicon*^+/+^ and *Rubicon*^−/−^ hearts after the surgery. The reason why the autophagic activity was not affected by Rubicon ablation remains to be elucidated, but it might be due to the inhibition of the signaling pathway upstream of Rubicon in the TAC-operated hearts. The fact that the level of hypertrophic responses was not different between both TAC-operated groups suggests that signaling of mTORC1, a master regulator of protein synthesis^15^, was activated to a similar extent to synthesize protein or cellular components for cardiac hypertrophy. As mTORC1 is also an autophagic suppressor^15^, its activation would have inhibited autophagosome formation at the same level in the two groups. Rubicon exists downstream of mTORC1 signaling in the autophagy cascade^5^. As a result of the suppression of the autophagic mechanism at the initiation step, Rubicon deficiency could not change the autophagic activity in our model.

Two markers of autophagic activity (LC3-II and p62) showed different responses to TAC; LC3-II was unchanged, but p62 was increased. The amount of p62 is regulated by both transcriptional regulation and post-translational degradation^16^. p62 participates in not only autophagic but also proteasomal degradation^17^. The increase in the protein level of p62 in response to TAC may have reflected not only autophagy activity but also the balance of protein synthesis and degradation.

A recent study reported the role of Rubicon in ischemia/reperfusion injury^8^. In that report, autophagic flux was increased in Rubicon-deficient hearts after ischemia/reperfusion. The role of Rubicon in the autophagic pathway might depend on experimental models such as the ischemia/reperfusion model in that study and a pressure-overload model in the heart as well as other tissues as described previously. The differences in the response to stress might increase the difficulty in selecting indications when Rubicon is applied to clinical treatment.

The result of the recycling assay suggests that in cardiomyocytes, Rubicon positively regulates $\upbeta$1 adrenergic receptor recycling under prolonged stimulation of its agonist. An increase in the expression level of Rubicon in the heart after TAC would be a pivotal phenomenon for the hypertrophic responses to downregulate endolysosomal degradation and to retain $\upbeta$1 adrenergic receptor on the cell membrane for maintaining $\upbeta$1 signaling. An important effector downstream of $\upbeta$1 adrenergic receptor signaling is calcium handling^18^. The pathway includes the activation of the adenylyl cyclases, resulting in increased cAMP levels. cAMP-dependent protein kinase (PKA) phosphorylates several calcium signaling proteins essential for cardiac function, such as L-type calcium channels, phospholamban, and ryanodine receptors. The dysregulation of calcium handling results in cardiomyocyte dysfunction and further reduction in responsiveness against $\upbeta$1 stimulation. As we did not evaluate calcium dynamics in this study, further investigation is required. The downregulation of $\upbeta$1 adrenergic receptor with the dysregulation of intracellular calcium handling in cardiomyocytes is one of the main causes of heart failure^19^. However, previous reports showed that an ablation of $\upbeta$1 adrenergic receptor did not affect or might protect against the progression of heart failure following TAC or myocardial infarction^20,21^. The absence of the receptor might lead to less toxic cAMP-PKA and cAMP-CaMKII signaling, resulting in improved cardiac function. We did not have any direct evidence to explain the apparent discrepancy regarding the role of $\upbeta$1 adrenergic receptor in failing hearts. The receptor was genetically ablated at an embryonic stage in the mice used in the knockout studies, while in our mouse model, $\upbeta$1 adrenergic receptor was only decreased after TAC. This could be one of reasons for the discrepancy between the knockout and our models.

Based on the results of the present study, it is suggested that Rubicon might have an important role in regulating the amount of $\upbeta$1 adrenergic receptor in cardiomyocytes. In our study, the level of intracellular $\upbeta$1 adrenergic receptor was decreased in Rubicon-knockdown cells. This result suggests that Rubicon knockdown-induced increase in the recycling of the receptor was dominantly caused by the upregulation of the degradation rather than endocytosis. However, there is still a possibility that increased endocytosis of $\upbeta$1 adrenergic receptor in Rubicon-knockdown cells may be involved in the phenomenon.

In this study, we reveal that Rubicon protects the heart from pressure overload. Rubicon is involved in the maintenance of the intracellular recycling of $\upbeta$1 adrenergic receptor, which might contribute to its cardioprotective effect. Medication with beta-blockers is an essential treatment for heart failure to normalize $\upbeta$1 adrenergic receptor signaling. The regulation of Rubicon could be a novel therapeutic target in heart failure.

## Methods

### Study approval

All experimental protocols were approved by the Animal Research Committee of Osaka University. All experiments were performed in accordance with the Guidelines for Animal Experiments of Osaka University and the Japanese Animal Protection and Management Law, and were carried out in accordance with the U.K. Animals (Scientific Procedures) Act 1986, and the associated guidelines, Directive 2010/63/EU for animal experiments. The authors complied with ARRIVE (Animal Research: Reporting of In Vivo Experiments) guidelines.

### Generation of cardiomyocyte-specific Rubicon-deficient mice

We crossed mice bearing a *Rubicon*^flox^ allele^6^ with transgenic mice expressing *Cre* recombinase under the control of $\mathrm{\alpha }$*-MHC* promoter^12^, to produce cardiomyocyte-specific Rubicon-deficient mice, *Rubicon*^flox/flox^;$\mathrm{\alpha }$*-MHC-Cre*^+^ (*Rubicon*^−/−^). Their *Rubicon*^flox/flox^;$\mathrm{\alpha }$*-MHC-Cre*^−^ (*Rubicon*^+/+^) littermates were used as controls. Only male mice were used in this study, to minimize any variability of baseline characteristics and phenotypes. The mice were given food and water ad libitum.

### Echocardiography and transverse aortic constriction

Echocardiography was performed on conscious mice using an ultrasonography equipped with a 15-MHz linear transducer (SONOS-4500, Philips Medical Systems). An M-mode echocardiogram of the midventricle was recorded at the level of the papillary muscles in the two-dimensional parasternal short-axis view. The following parameters were obtained to assess LV size and function: heart rate (HR), end-diastolic interventricular septal and LV posterior wall thickness (IVSd and LVPWd), end-diastolic and end-systolic internal dimensions of LV (LVDd and LVDs) and fractional shortening of LV dimension (FS) and LV mass. FS and LV mass were calculated as 100 $\times$ (LVDd—LVDs)/LVDd and 1.05 $\times$ [(LVDd + IVSd + LVPWd)^3^—(LVDd)^3^], respectively.

The 8‒11-week-old male mice underwent TAC surgery using 26-gauge needles^22^. Non-invasive measurements of blood pressure were performed on mice anaesthetized with 2.5% avertin using a blood pressure monitor for mice (Model MK-2000, Muromachi Kikai), according to the manufacturer’s instructions as previously described^22^.

### Western blot analysis

Total protein homogenates from the LV or lysate of NRCM underwent western blot analysis. The antibodies used in this study were follows: a monoclonal rabbit antibody to mouse Rubicon (#8465, Cell Signaling Technology), a monoclonal rabbit antibody to rat Rubicon (21444-1-AP, Proteintech), a monoclonal mouse antibody to glyceraldehyde-3-phosphate dehydrogenase (GAPDH) (016-25523, FUJIFILM Wako Pure Chemical Corporation), a polyclonal rabbit antibody to p62 (PM045, MBL), a polyclonal rabbit antibody to LC3 (#2775, Cell Signaling Technology), a polyclonal rabbit antibody to $\upbeta$1 adrenergic receptor (ab3442, abcam), a monoclonal mouse antibody to phospholamban (ab2865, abcam), and a polyclonal rabbit antibody to phosphorylated phospholamban (07-052, Sigma Aldrich). The antibody to $\upbeta$1 adrenergic receptor was validated by western blotting analysis of HA-tagged mouse $\upbeta$1 adrenergic receptor (Supplementary Fig. S2). After incubation with secondary antibody, the blot was developed with ImmunoStar Zeta or ImmunoStar LD reagent (FUJIFILM Wako Pure Chemical Corporation). ImageQuantTL (version 7.0, Cytiva) was used for quantitative analysis to evaluate protein expression levels.

### Histological analysis

Heart samples were excised and fixed in 10% neutral buffered formalin. After paraffin embedding, the samples were cut into 5-$\upmu$m sections. Serial sections were stained with hematoxylin and eosin or AZAN-Mallory staining^23^ and observed using a microscopy, BZ-9000 (Keyence). Quantitative analysis of the cardiomyocyte cross-sectional area was examined by tracing the outline of 100–200 myocytes in each section and that of fibrosis was examined in the whole area of sections using ImageJ (version 1.51j8, National Institutes of Health)^23^.

TUNEL assay was performed using In Situ Cell Death Detection Kit, Fluorescein (Roche) and the sections were mounted with VECTASHIELD with DAPI (Vector Laboratories). Fluorescent signals were observed using a fluorescent microscopy (FV-1000, Olympus). The number of TUNEL-positive nuclei and total nuclei of cardiomyocytes was counted.

For immunohistochemical analysis, frozen heart sections (5 $\upmu$m) were fixed in buffered 4% paraformaldehyde. The primary antibodies (anti-mouse CD45 [MAB114, R&D Systems], CD68 [MCT1957T, Bio-Rad], Ly6G/6C [550291, BD Pharmacy], CD3 [ab16669, Abcam], avidin-peroxidase (Vectastain Elite ABC Kit; Vector Laboratories) and ImmPACT DAB Peroxidase Substrate Kit (Vector Laboratories) were used, followed by counterstaining with hematoxylin, as described previously^22^.

### Real-time quantitative reverse transcription polymerase chain reaction

Total RNA was isolated from the LV using TRIzol reagent (Thermo Fisher Scientific). The mRNA expression levels were determined by quantitative reverse transcription polymerase chain reaction (PCR) using MultiScribe Reverse Transcriptase (Thermo Fisher Scientific) for reverse transcription and Platinum Quantitative PCR SuperMix-UDG (Thermo Fisher Scientific) for the quantitative PCR^24^ with PCR primers and probes from Applied Biosystems as follows: Assay ID: Mm01255747_g1 for *Nppa*, Assay ID: Mm00435304_g1 for *Nppb*, Assay ID: Mm01165187_m1 for *Co1a2*, Assay ID: Mm99999064_m1 for interleukin (IL)-6 (*Il6*), Assay ID: Mm00443260_g1 for tumor necrosis factor (TNF)-α (*Tnfa*) and *Gapdh*, Assay ID: Mm99999915_g1. All data were normalized to the *Gapdh* mRNA comparative threshold (Ct) value and are expressed as the fold increase over the control group.

### Hemodynamic assessment of cardiac function in vivo

Three days after TAC, the mice were anesthetized with 4% isoflurane for induction and placed on a thermally controlled surgical table. After intratracheal intubation, the intubation cannula was connected to a respirator (SAR-830, CWE) and then the isoflurane concentration was decreased to 1.0‒1.5%. Thoracotomy was performed and the pericardium was removed from the heart. A 1-French Millar catheter was inserted into LV from the apex. After a 10-min stabilization period, the inferior vena cava was cannulated and connected to a microinjection pump (KDS 100, Muromachi Kikai) for the infusion of 1, 2 and 5 $\upmu$g/kg/min of dobutamine. The LV pressure was digitized and processed by a computer system (PowerLab/8S with Chart 5 software, AD instruments). As cannulation to the inferior vena cava affects the preload resulting in decreased cardiac parameters^25,26^, the data after dobutamine infusion were normalized with those at 1 $\upmu$g/kg/min dobutamine.

### Isolation of NRCMs and adenoviral infection

We isolated NRCMs from the ventricles of 1‒2-day-old Wistar rats and plated them as previously reported^2^. After 48-h incubation, the cells were infected with adenovirus expressing shRNA targeted to Rubicon at 30 multiplicity of infection (MOI). The target-specific shRNA duplexes were designed from the open reading frame of rat Rubicon mRNA. The selected sequence was validated with a BLAST search. Finally, a shRNA fragment was inserted into the pAd-CMV/V5-DEST Vector (ThermoFisher Scientific). The negative control shRNA was constructed using pAd/CMV/V5-GW/lacZ control plasmid (ThermoFisher Scientific). The plasmids were transfected into HEK293 cells to obtain the adenoviruses. The adenoviruses were amplified by infection into HEK293 cells, purified using Adenovirus Mini Purification Virakit (VIRAPUR), and titrated using Adeno-X Rapid Titer Kit (Clontech).

### Autophagic flux assay in NRCM with knockdown of Rubicon

Forty-eight hours after being seeded, NRCMs were infected with adenovirus harboring Rubicon shRNA at 30 MOI. At 72 h after infection, the cells were treated with 100 nM bafilomycin A1 or DMSO for 24 h. The lysate was used for western blot analysis.

### Recycling assay

We performed the recycling assay as previously reported^27^. NRCMs were seeded on 18-mm glass slides coated with cell matrix I-C (KP-4020, KURABO INDUSTRIES). After 48 h, the cells were infected with adenovirus harboring Rubicon shRNA at 30 MOI. At 72 h after infection, the non-stimulated NRCMs were blocked for 15 min in the presence of 10% normal donkey serum in DMEM at 37 °C. Anti-$\upbeta$1 adrenergic receptor antibody (NB600-978, Novus biologicals) was then added at a dilution of 1:100 in DMEM with 1% donkey serum and the cells were incubated at 37 °C for one hour. The antibody-containing medium was aspirated and the cells were washed with acid-cold MEM, pH 2.0 for two times and in cold PBS once. The NRCMs were returned to 10% donkey serum in DMEM for one hour. Alexa Fluor 488-labelled anti-goat IgG antibody (A32814, ThermoFisher Scientific) was diluted 1:1000 in 1% donkey serum in DMEM, and added to the cells for one hour at 37 °C to label the recycled receptors. The cells were subsequently acid-washed as described above, and then fixed in 4% paraformaldehyde in PBS for 15 min at 37 °C. The cells were washed with PBS containing 100 mM glycine twice and with PBS once, and then mounted with VECTASHIELD with DAPI (Vector Laboratories).

Following the above protocol, the fluorescent signal from the recycled receptors was intracellular and readily determined by fluorescent microscopy (FV-1000, Olympus). The recycling was quantified by calculating the summation of Alexa Fluor 488 pixels divided by the total number of cells. The images were analyzed and quantified with ImageJ (Version 1.51j8, National Institutes of Health).

### Intracellular $\upbeta$1 adrenergic receptor staining

NRCMs were seeded on 18-mm glass slides coated with cell matrix I-C (KP-4020, KURABO INDUSTRIES). After 48 h, the cells were infected with adenovirus harboring Rubicon shRNA at 30 MOI. At 72 h after infection, the cells were fixed in 4% paraformaldehyde in DMEM for 15 min at 37 °C, then permeabilized with 0.3% Tween-20 in PBS for 10 min at room temperature and blocked with 2% bovine serum albumin in PBS for one hour at 37 °C. Anti-$\upbeta$1 adrenergic receptor antibody (NB600-978, Novus biologicals) was added at a dilution of 1:100 in 2% bovine serum albumin/PBS and the cells were incubated at 4 °C for over-night. After wash with PBS for three times, the cells were incubated with Alexa Fluor 488-labelled anti-goat IgG antibody (A32814, ThermoFisher Scientific) diluted at 1:500 in 2% bovine serum albumin/PBS for one hour at room temperature and then mounted with VECTASHIELD with DAPI (Vector Laboratories).

Following the above protocol, the intracellular fluorescent signal was determined by fluorescent microscopy (FV-1000, Olympus) and quantified. The images were analyzed and quantified with ImageJ (Version 1.51j8, National Institutes of Health).

### Statistical analysis

Data are shown as the mean ± s.e.m. GraphPad Prism (version 8.4.3, GraphPad Software, La Jolla, California) was used for statistical analysis. Student’s *t*-test was used for 2-group comparison; one-way analysis of variance (ANOVA) followed by Tukey’s post hoc test or two-way ANOVA followed by Bonferroni’s post hoc test was used for multiple comparisons. P < 0.05 was considered statistically significant.

## Supplementary Information

Supplementary Information.

## Abbreviations

$\mathrm{\alpha }$-MHC: Alpha-myosin heavy chain
FS: Fractional shortening of left ventricular dimension
HR: Heart rate
IVSd: End-diastolic interventricular septal wall thickness
LC3: Microtubule-associated protein 1 light chain 3
LVDd: End-diastolic internal dimension of the left ventricle
LVDs: End-systolic internal dimension of the left ventricle
LVPWd: End-diastolic left ventricle posterior wall thickness
Rubicon: Run domain Beclin-1-interacting and cysteine-rich domain-containing protein
TAC: Transverse aortic constriction
TUNEL: Terminal deoxynucleotidyl transferase-mediated dUTP-biotin nick end labeling
