# Supplementary material for: Rubicon-regulated beta-1 adrenergic receptor recycling protects the heart from pressure overload
Source: Sci Rep. 2022 Jan 7;12:41. doi: 10.1038/s41598-021-03920-6 (PMC8741968; doi:10.1038/s41598-021-03920-6)
Supplement: Supplementary file 1 — Supplementary Information. [file 41598_2021_3920_MOESM1_ESM.pdf]

Supplementary Information

Rubicon-regulated β1 adrenergic receptor recycling protects the heart from pressure overload

Yasuhiro Akazawa, Manabu Taneike, Hiromichi Ueda, Rika Kitazume-Taneike,
Tomokazu Murakawa, Ryuta Sugihara, Hiroki Yorifuji, Hiroki Nishida, Kentaro Mine,
Ayana Hioki, Shigemiki Omiya, Hiroyuki Nakayama, Osamu Yamaguchi,
Tamotsu Yoshimori, Yasushi Sakata, Kinya Otsu

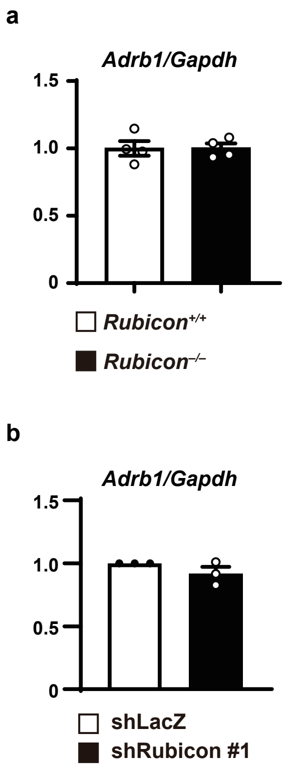

Supplementary Fig. S1.

Evaluation of *Adrb1* gene expression level.

(a) mRNA expression of *Adrb1* in *Rubicon*$^{-/-}$ mouse left ventricles. *Gapdh* mRNA was used as the loading control. The average value for *Rubicon*$^{+/+}$ hearts was set to 1. N = 4. Student' s *t*-test was used for analysis. Open and closed bars indicate, *Rubicon*$^{+/+}$ and *Rubicon*$^{-/-}$, respectively.

(b) mRNA expression of *Adrb1* in Rubicon knockdowned neonatal rat cardiomyocytes. *Gapdh* mRNA was used as the loading control. The value for shLacZ infected cells in each experiment was set equal to 1. N = 3. Student' s *t*-test was used for analysis. Open and closed bars indicate, shLacZ and shRubicon, respectively.

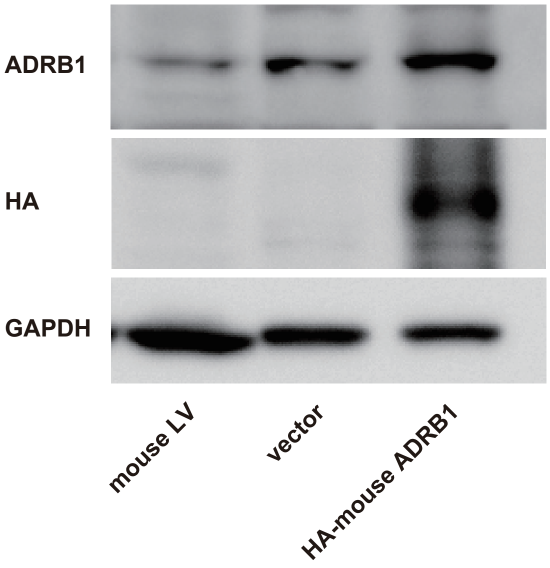

Supplementary Fig. S2. Validation of anti-ADRB1 antibody.

Lysate from wild-type mouse left ventricule was subjected to immunoblotting with the indicated antibodies together with lysates from HEK293A cells transfected with empty vector or HA-mouse ADRB1 .

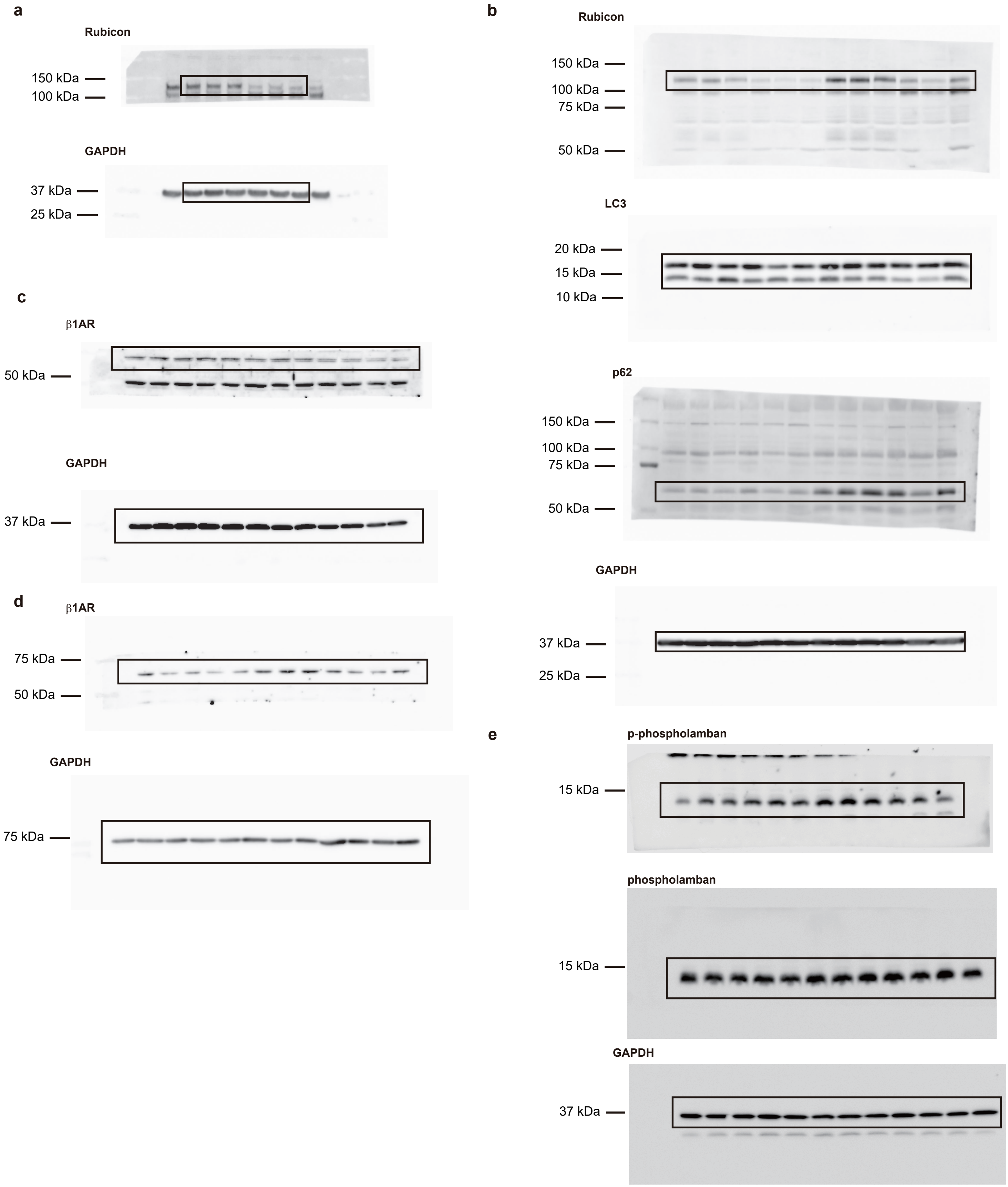

Supplementary Fig. S3. Full-length blots used for Fig. 1 and 3

(a) Blots for Fig. 1a. (b) Blots for Fig. 3a. (c) Blots for Fig. 3b. (d) Blots for Fig. 3c.

(e) Blots for Fig. 3e. Area shown in figures are indicated by boxes.

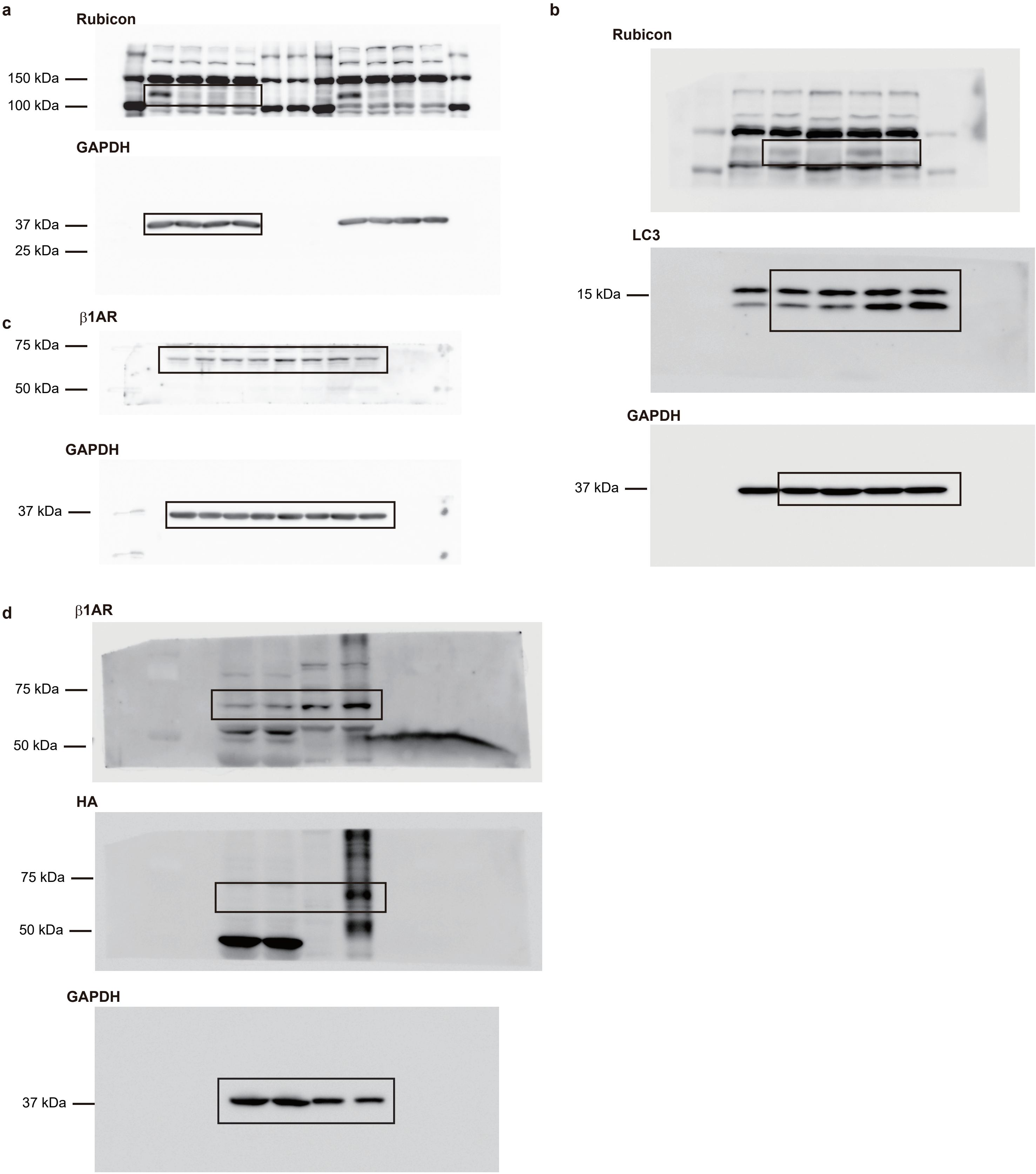

Supplementary Fig. S4. Full-length blots used for Fig. 4

(a) Blots for Fig. 4a. (b) Blots for Fig. 4b. (c) Blots for Fig. 4c. (d) Blots for Supplementary Fig. S2. Area shown in figures are indicated by boxes.
